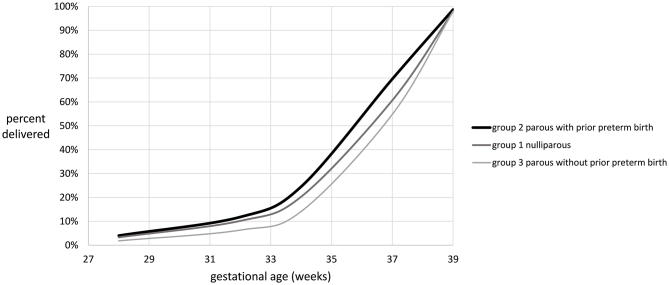# Effects of parity on preterm delivery in twin gestations conceived with in vitro fertilization

**DOI:** 10.1016/j.xfre.2023.01.005

**Published:** 2023-01-21

**Authors:** Michael S. Awadalla, Wael H. Salem, Jacqueline R. Ho, Victoria K. Cortessis, Ali Ahmady, Richard J. Paulson

**Affiliations:** a Division of Reproductive Endocrinology and Infertility, Department of Obstetrics and Gynecology, Keck School of Medicine, University of Southern California, Los Angeles, California; b Department of Population and Public Health Sciences, Keck School of Medicine, University of Southern California, Los Angeles, California

**Keywords:** In vitro fertilization, twin gestation, preterm delivery, parity

## Abstract

**Objective:**

To determine the relationship between prior obstetrical history and gestational age at delivery in a twin pregnancy.

**Design:**

Retrospective cohort study using the United States Society for Assisted Reproductive Technology Clinic Outcomes Reporting System database.

**Setting:**

Clinic-based data.

**Patient(s):**

Patients undergoing in vitro fertilization (IVF) in the United States with live delivery of twins.

**Intervention(s):**

None.

**Main outcome measure(s):**

The main outcome measures are median gestational age at delivery and rate of preterm delivery (before 37 weeks).

**Result(s):**

The median gestational age at delivery of IVF-conceived twins was 36.3 (interquartile rate 34.4, 37.6) weeks for nulliparous women, 35.9 (34.0, 37.1) weeks for parous women with a prior preterm birth, and 36.7 (35.1, 37.7) weeks for parous women without a prior preterm birth. The rate of preterm delivery was 61% for nulliparous women, 70% for parous women with a prior preterm birth, and 55% for parous women without a prior preterm birth.

**Conclusion(s):**

Parous women without a history of preterm delivery had lower rates of preterm delivery in a subsequent twin pregnancy than nulliparous women. Nulliparous women had lower rates of preterm delivery compared with parous women with a history of preterm delivery.

Despite recent trends in practice patterns toward the performance of more single embryo transfers, multiple embryo transfer is still often performed to maintain pregnancy rates. In 2018, in the United States, approximately 13% of deliveries after in vitro fertilization (IVF) with autologous oocytes were twins or higher-order multiples (1). This equates to approximately 23% of infants born from IVF being exposed to the risk of multiple gestations. Although twin pregnancies are more common after multiple embryo transfer, the twin rate after a single embryo transfer is still 1–2%. Knowledge of how obstetrical history affects the risk of preterm delivery in a twin pregnancy is important for patient counseling and for determining how many embryos can be safely transferred in a given patient. The United States Society for Assisted Reproductive Technology Clinic Outcomes Reporting System (SART CORS) database contains IVF data including embryo transfer and delivery dates as well as obstetrical history. This is an ideal database to evaluate the effect of prior obstetrical history on twin pregnancy outcomes.

The two most common perinatal morbidities with twin gestations are preterm delivery and low birth weight. Twins on average are born at 35.3 weeks gestation compared with 39.1 weeks for singletons (2). The mean birth weight for a twin is 2,347 grams compared with 3,358 grams for a singleton (2). Previous studies have shown that twin pregnancies in multiparous women without a history of preterm delivery are more likely to deliver at later gestational ages than twin pregnancies in nulliparous women or multiparous women with a previous preterm delivery (3, 4, 5). A recent study found that even a history of early-term singleton delivery was associated with preterm birth in a subsequent twin pregnancy (6). None of these previous studies focused on an IVF population where precise pregnancy dating is known. Since attempts to prevent preterm birth in twin gestations have had very limited success, precise knowledge of the risk of preterm delivery for a twin pregnancy is important (7).

The primary objective of this study was to determine the relationship between parity and length of gestation in twin gestation. The secondary objectives were to determine the relationship between parity, history of preterm delivery, and length of gestation and fetal weight in a subsequent twin gestation.

## Materials and methods

### Study Population and Data

The SART CORS database contains comprehensive data from >90% of all clinics performing assisted reproductive technology (ART) cycles in the United States. The data were collected through voluntary submission, verified by SART, and then reported to the Centers for Disease Control and Prevention in compliance with the Fertility Clinic Success Rate and Certification Act of 1992 (Public Law 102-493). The SART maintains HIPAA-compliant business associate agreements with reporting clinics. In 2004, after a contract change with the Centers for Disease Control and Prevention, SART gained access to the SART CORS data system for research purposes. The data in the SART CORS are validated annually, with select clinics having on-site visits for chart review based on an algorithm for clinic selection (8). During each visit, data reported by the clinic were verified with information recorded in patients’ charts (8). In 2012, records for 2,045 cycles at 35 clinics were randomly selected for full validation, along with 238 egg or embryo banking cycles. The full validation included a review of 1,318 cycles for which pregnancy was reported. Among the nondonor cycles, 331 were multiple-fetus pregnancies. Ten out of 11 data fields selected for validation were found to have discrepancy rates of ≤5%. The exception was the diagnosis field, which, depending on the diagnosis, had a discrepancy rate between 2.1% and 9.2% (8).

**Figure 1:**
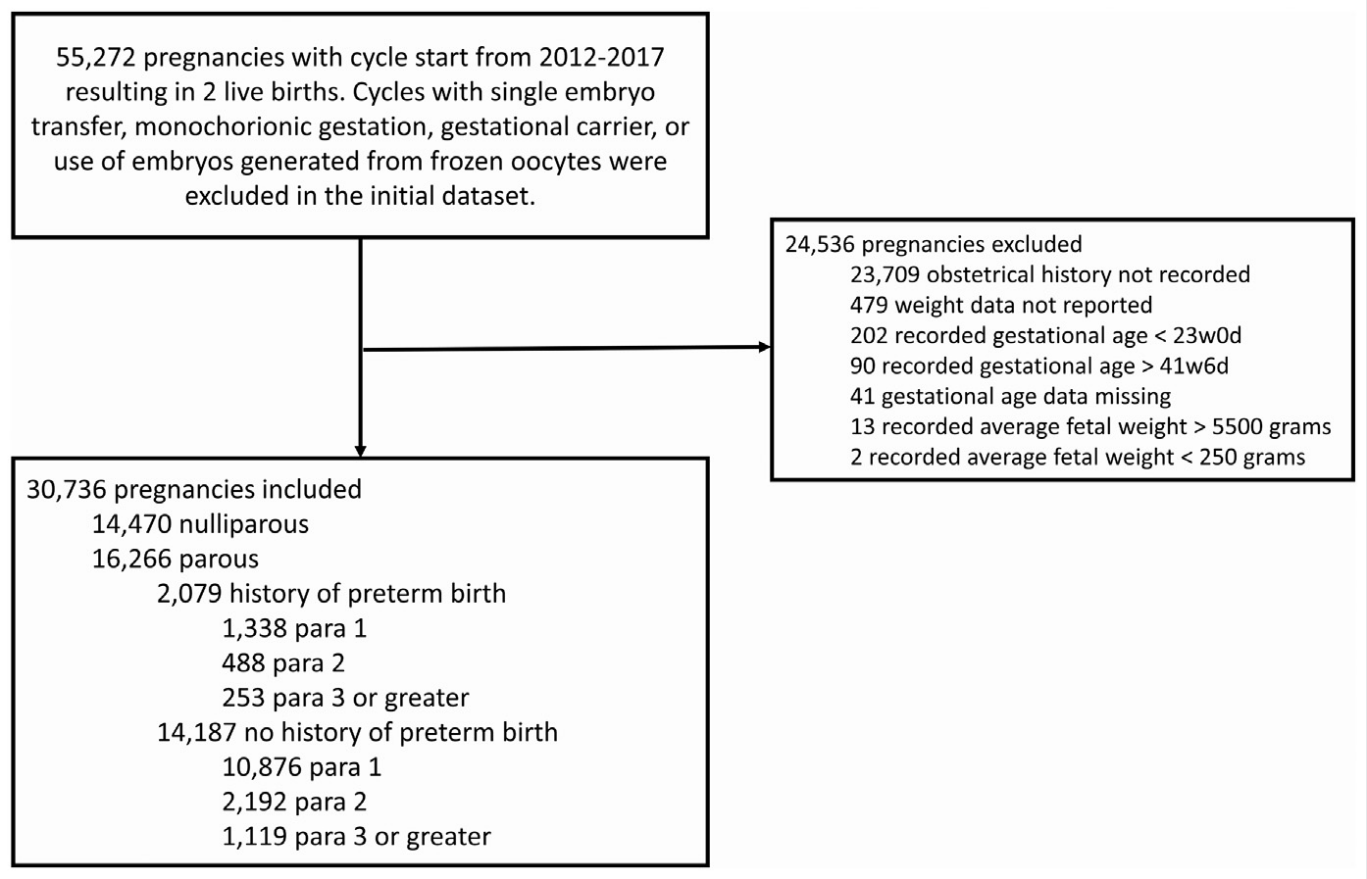
Flow diagram of included and excluded cycles

This study included patients aged 20 to 50 with a live twin delivery in the SART CORS database with cycles from 2012 to 2017. Cycles were excluded if there was a single embryo transfer, monochorionic gestation, use of a gestational carrier, or pregnancy after a transfer of an embryo created from a frozen oocyte. The initial dataset obtained included 55,272 pregnancies. A total of 24,536 pregnancies were excluded for unrecorded gravidity or parity, fetal weight data not recorded, gestational age data not recorded, recorded gestational age less than 23 weeks 0 days or greater than 41 weeks 6 days, or average fetal weight less than 250 grams or greater than 5500 grams. Data from the remaining 30,736 pregnancies were included in the primary analysis (Figure 1 and Table 1). The SART CORS dataset contains data on the history of preterm delivery as a dichotomous variable but does not contain information on the number of prior preterm births or the degree of prematurity. This study was approved by the University of Southern California institutional review board (HS-16-00396) and the SART research committee.

**Table 1:** Baseline demographic and clinical characteristics.

Demographics and clinical characteristics	Nulliparous n = 14,470	Parous n = 16,266	*P* value
Maternal age (SD)	34.8 (5.1)	35.3 (4.7)	<.01
Maternal BMI kg/m^2^ (SD)∗	25.8 (5.7)	25.9 (5.6)	
Maternal BMI kg/m^2^ [IQR]∗	24.4 [21.8, 28.5]	24.6 [21.8, 28.7]	.02
Fetal sex			
Female/female, n (%)	3,443 (23.8)	4,164 (25.6)	<.01
Female/male†, n (%)	7,285 (50.3)	7,686 (47.3)	
Male/male, n (%)	3,742 (25.9)	4,416 (27.1)	
Parity	0	1.4	
Prior term birth, n (%)	0	14,810 (91.0)	
Prior preterm birth, n (%)	0	2,079 (12.8)	
Prior SAB, n (%)	9,777 (67.6)	6,181 (38.0)	<.01
Autologous oocytes, n (%)	11,733 (81.1)	13,839 (85.1)	<.01
Donor oocytes, n (%)	2,592 (17.9)	2,265 (13.9)	<.01
Donor embryo, n (%)	151 (1.0)	169 (1.0)	1.00
Fresh embryo, n (%)	7,941 (54.9)	8,295 (51.0)	<.01
Frozen embryo, n (%)	6,552 (45.3)	7,987 (49.1)	<.01
No. of embryos transferred	2.13	2.12	n/a
2, n (%)	13,020 (90.0)	14,607 (89.8)	.02
3, n (%)	1,141 (7.9)	1,377 (8.5)	
4, n (%)	244 (1.7)	218 (1.3)	
≥ 5, n (%)	65 (0.4)	64 (0.4)	
No.of fetal heartbeats	2.02	2.02	n/a
2, n (%)	14,164 (97.9)	15,968 (98.2)	.20
3, n (%)	280 (1.9)	274 (1.7)	
4, n (%)	26 (0.2)	24 (0.1)	
PGT all or some embryos, n (%)	1,496 (10.3)	1,507 (9.3)	.002
Maternal ethnicity			
White, n (%)	6,580 (45.5)	7,897 (48.5)	<.01
Asian, n (%)	1,153 (8.0)	919 (5.6)	
Hispanic, n (%)	686 (4.7)	939 (5.8)	
African American, n (%)	665 (4.6)	724 (4.5)	
American Indian Alaskan Native, n (%)	18 (0.1)	12 (0.1)	
Native Hawaii other pacific, n (%)	19 (0.1)	21 (0.1)	
Multiple, n (%)	216 (1.5)	180 (1.1)	
Unknown, n (%)	3,922 (27.1)	4,325 (26.6)	
Not Asked, n (%)	1,192 (8.2)	1,230 (7.6)	
Refused, n (%)	19 (0.1)	19 (0.1)	
Infertility diagnosis‡			
Male factor, n (%)	4,796 (33.1)	5,835 (35.9)	<.01
Diminished ovarian reserve, n (%)	3,913 (27.0)	3,673 (22.6)	<.01
Polycystic Ovary Syndrome, n (%)	2,454 (17.0)	2,791 (17.2)	.64
Tubal factor, n (%)	2,111 (14.6)	3,034 (18.7)	<.01
Ovulatory Disorder, n (%)	2,082 (14.4)	2,202 (13.5)	.03
Unexplained infertility, n (%)	1,998 (13.8)	1,910 (11.7)	<.01
Endometriosis, n (%)	1,369 (9.5)	1,396 (8.6)	<.01
Uterine factor, n (%)	676 (4.7)	628 (3.9)	<.01
Recurrent pregnancy loss, n (%)	133 (0.9)	100 (0.6)	<.01
Other, n (%)	2,112 (14.6)	2,377 (14.6)	.97

*Note:* Data are reported as mean, mean (SD), median [IQR], or n (%). *P* values are reported using t-test for normally distributed continuous data, Wilcoxon rank-sum test for nonnormally distributed continuous data, Fisher’s exact test for dichotomous and categorical data with fewer than five categories, and Chi-square test for categorical data with greater than five categories. BMI = body mass index; PGT = preimplantation genetic testing

∗ BMI data available for 82% of sample.

† 93 instances of unknown fetal sex recorded as female/male.

‡ Since multiple diagnosis are recorded for some patients the sum of the percentages exceeds 100%.

### Statistical Methods

The gestational age at delivery and average birth weight were nonnormally distributed. The unadjusted analysis of outcomes was performed using Fisher’s exact test for categorical data and the Wilcoxon rank-sum test for continuous data. There were 93 instances of unknown fetal sex, and these were recorded as male/female because this was the most frequent combination.

Two logistic regression models were built, one for the rate of preterm delivery and the other for rate of low average birth weight (Stata version 16.1, StataCorp, College Station, TX). We used the methods described by Hosmer and Lemeshow for the purposeful selection of covariates (9). Demographic and clinical characteristics that differed (*P*<.05) between nulliparous and parous groups were considered for evaluation as confounders. These characteristics included age, fetal sex, prior spontaneous abortion, use of autologous oocytes, use of one or more fresh embryos, the number of fetal heartbeats detected, use of preimplantation genetic testing, maternal ethnicity, and infertility diagnosis (Table 1). Covariates that were independently associated with the outcomes of preterm delivery or low average birth weight in nulliparous women with a *P*< .20 (and an absolute difference of at least 1% between nulliparous and parous groups for ethnicity and infertility) were included in the preliminary model as shown in Supplemental Table 1(available online). Age was included in the preliminary model for low birth weight although it was not statistically significant since a previous study has shown potential clinical significance (4). For both models, we used a number of fetal heartbeats in the model and omitted the number of embryos transferred because fetal heartbeats are more clinically significant and including both would introduce collinearity. The body mass index was excluded from both analyses because this data was only available for 82% of pregnancies and the difference between groups was not clinically significant.

The preliminary model consisted of all the variables identified on univariate analysis that differed between nulliparous and parous groups and were associated with preterm delivery or low birth weight in nulliparous patients. We then fit multivariable models (one model for preterm delivery and another model for low birth weight) containing all the identified variables and assessed the significance of each variable in the multivariable model. The final model was determined by removing variables that were insignificant in the multivariable model based on a *P*>.05 and no expected clinical significance.

For preterm delivery (before 37 weeks), there were no changes from the preliminary to the final model. All predictors were statistically significant except for the use of preimplantation genetic testing (PGT) and White ethnicity, which were left in the model because of potential clinical significance. The interaction between parity and age was not significant and was not included in the final model. The final model (Table 2 and Supplemental Table 1) for preterm delivery included age as a continuous variable and the following as dichotomous variables: parous, use of autologous oocytes, use of one or more fresh embryos, the number of fetal heartbeats (2 or more than 2), use of PGT, White or not White, Asian or not Asian, and history of unexplained infertility. This same model was used in our secondary analysis to compare nulliparous, parous with a history of preterm delivery, and parous without a history of preterm delivery (Table 3). We used this model to analyze delivery before 39 weeks, before 37 weeks, before 34 weeks, before 32 weeks, and before 28 weeks.

**Table 2:** Primary analysis of obstetrical outcomes based on parity.

Demographics and clinical characteristics	Nulliparous (reference) n = 14,470	Parous n = 16,266	Rate difference (95% CI)	OR (95% CI)	Unadjusted *P* value	Adjusted OR (95% CI)	Adjusted *P* value
Birth weight in grams (mean)	2344 (564)	2497 (533)					
Birth weight in grams (median)	2424 [2055, 2722]	2566 [2226, 2849]			< .01		
Birth weight >4000g, n (%)	13 (0.1)	15 (0.1)	0.00 (-0.07-0.07)	1.03 (0.49-2.16)	1.00	1.12 (0.53-2.36)	.77
Low birth weight, <2500g, n (%)	8,215 (56.8)	7,293 (44.8)	11.9 (10.8-13.0)	0.62 (0.59-0.65)	< .01	0.63 (0.60-0.66)	< .01
Very low birth weight, <1500g, n (%)	1,216 (8.4)	826 (5.1)	3.3 (2.8-3.9)	0.58 (0.53-0.64)	< .01	0.58 (0.53-0.64)	< .01
Gestational age in wk (mean)	35.5 (2.9)	35.9 (2.6)					
Gestational age in wk (median)	36.3 [34.4, 37.6]	36.6 [35.0, 37.6]			< .01		
Delivery before 39 wk, n (%)	14,170 (97.9)	15,938 (98.0)	-0.06% (-0.37 to 0.26)	1.03 (0.88-1.21)	.75	1.03 (0.88-1.21)	.69
Delivery before 37 wk, n (%)	8,784 (60.7)	9,223 (56.7)	4.0 (2.9-5.1)	0.85 (0.81-0.89)	< .01	0.86 (0.82-0.90)	< .01
Delivery before 34 wk, n (%)	2,934 (20.3)	2,504 (15.4)	4.9 (4.0%-5.7)	0.72 (0.67-0.76)	< .01	0.73 (0.68-0.77)	< .01
Delivery before 32 wk, n (%)	1,482 (10.2)	1,138 (7.0)	3.2 (2.6%-3.9)	0.66 (0.61-0.71)	< .01	0.67 (0.62-0.73)	< .01
Delivery before 28 wk, n (%)	477 (3.3)	340 (2.1)	1.2 (0.8-1.6)	0.63 (0.54-0.72)	< .01	0.63 (0.55-0.73)	< .01

*Note:* Data are reported as mean (SD), median [IQR] or n (%). Unadjusted P values are reported using the Wilcoxon rank-sum test for nonnormally distributed continuous data and Fisher’s exact test for dichotomous data. Adjusted *P* values are reported using logistic regression.

**Table 3:** Secondary analysis of obstetrical outcomes based on parity and history of preterm birth.

Demographics and clinical characteristics	Group 1 nulliparous n = 14,470	Group 2 parous with prior preterm birth n = 2,079	Group 3 parous without prior preterm birth n = 14,187	*P* value Groups 1 and 2	Adjusted *P* value group 1 vs 2	*P* value group 1 vs 3	Adjusted *P* value group 1 vs 3	*P* value group 2 vs 3	Adjusted *P* value group 2 vs 3
Birth weight in grams (mean)	2344 (564)	2346 (579)	2519 (523)						
Birth weight in grams (median)	2424 [2055, 2722]	2424 [2041, 2736]	2580 [2254, 2863]	.79		< .01		< .01	
Birth weight >4000g, n (%)	13 (0.1)	1 (0.0)	14 (0.1)	1.00	.68	.85	.66	.71	.52
Low birth weight, <2500g, n (%)	8,215 (56.8)	1,162 (55.9)	6,131 (43.2)	.45	.91	< .01	< .01	< .01	< .01
Very low birth weight, <1500g, n (%)	1,216 (8.4)	175 (8.4)	651 (4.6)	.97	.96	< .01	< .01	< .01	< .01
Gestational age in wk (mean)	35.5 (2.9)	35.1 (2.9)	36.0 (2.5)						
Gestational age in wk (median)	36.3 [34.4, 37.6]	35.9 [34.0, 37.1]	36.7 [35.1, 37.7]	< .01		< .01		< .01	
Delivery before 39 wk , n (%)	14,170 (97.9)	2,051 (98.7)	13,887 (97.9)	.03	.04	.84	.86	.02	.02
Delivery before 37 wk , n (%)	8,784 (60.7)	1,448 (69.6)	7,775 (54.8)	< .01	< .01	< .01	< .01	< .01	< .01
Delivery before 34 wk , n (%)	2,934 (20.3)	509 (24.5)	1,995 (14.1)	< .01	< .01	< .01	< .01	< .01	< .01
Delivery before 32 wk , n (%)	1,482 (10.2)	246 (11.8)	892 (6.3)	.03	.02	< .01	< .01	< .01	< .01
Delivery before 28 wk , n (%)	477 (3.3)	83 (4.0)	257 (1.8)	.10	.08	< .01	< .01	< .01	< .01

*Note:* Data are reported as mean (SD), median [IQR] or n (%). Unadjusted *P* values are reported using the Wilcoxon rank-sum test for nonnormally distributed continuous data and Fisher’s exact test for dichotomous data. Adjusted *P* values are reported using logistic regression.

For low average birth weight (< 2500 grams), there were also no changes from the preliminary to the final model. All predictors were statistically significant except for the use of PGT, diminished ovarian reserve, and unexplained infertility. These were left in the model because of potential clinical significance. The interaction between parity and age was not significant and was not included in the final model. The final model (Table 2 and Supplemental Table 1) for low average birth weight included age as a continuous variable and the number of male fetuses (0, 1, or 2) as an ordinal variable. The following dichotomous variables were included: parous, use of autologous oocytes, use of one or more fresh embryos, the number of fetal heartbeats (2 or more than 2), use of PGT, White or not White, Asian or not Asian, male factor infertility, diminished ovarian reserve, tubal factor, and unexplained infertility. This same model was used in our secondary analysis of parity (Table 3). We used this model for the analysis of average fetal weight (assessed as > 4000 grams, < 2500 grams, and < 1500 grams).

Hosmer and Lemeshow overall goodness of fit tests did not show evidence of poor fit for predicting the risk of preterm delivery (*P*=.16) or low average birth weight (*P*= .24). A *P*<.01 was considered statistically significant for comparisons of outcomes between nulliparous and parous groups.

## Results

### Primary Outcomes

The median gestational age at delivery for nulliparous women was 36.3 weeks compared with 36.6 weeks for parous women (*P*<.01, Table 2). The median birth weight for nulliparous women was 2,424 grams compared with 2,566 grams for parous women (*P*<.01, Table 2).

The preterm delivery rate (before 37 weeks) was 60.7% for nulliparous women compared with 56.7% for parous women (adjusted odds ratio [aOR] 0.86, *P* < .01, Table 2). The preterm delivery rate before 32 weeks was 10.2% for nulliparous women compared with 7.0% for parous women (aOR 0.67, *P* < .01, Table 2).

The rate of low birth weight (less than 2500 grams) was 56.8% for nulliparous women compared with 44.8% for parous women (aOR 0.63, *P* < .01, Table 2). The rate of very low birth weight (less than 1500 grams) was 8.4% for nulliparous women compared with 5.1% for parous women (aOR 0.58, *P* < 0.01, Table 2).

### Secondary Outcomes

Our secondary analysis compared birth weight and gestational age between nulliparous women, parous women with prior preterm birth, and parous women without prior preterm birth. The preterm delivery rate (before 37 weeks gestation) was 60.7% for nulliparous women, 69.6% for parous women with a prior preterm birth, and 54.8% for parous women without a prior preterm birth (adjusted *P* < .01 for all comparisons). The rate of low birth weight (less than 2500 grams) was 56.8% for nulliparous women, 55.9% for parous women with a prior preterm birth, and 43.2% for parous women without a prior preterm birth. There was no significant difference in the low-birth-weight rate between nulliparous and parous women with prior preterm birth (Table 3 and Supplemental Figure 1, [available online]).

We report obstetrical outcomes of parous women based on parity number in Supplemental Table 2. Outcomes for pregnancies medically or spontaneously reduced to twins are provided in Supplemental Table 3.

## Discussion

There are some differences in gestational age and birth weight based on obstetrical history, but these are small compared with the increased risk of preterm delivery in twins compared with singletons. Parous women with twins delivered at a median gestational age of 36.6 weeks compared with 36.3 weeks for nulliparous women. Parous women with a history of prior preterm birth delivered at a median gestational age of 35.9 weeks compared with 36.7 weeks for parous women without prior preterm birth (Table 3). Although some might consider these differences clinically significant when determining who is a candidate for multiple embryo transfer, others may consider them insignificant given that singletons are delivered at a mean gestational age of 39.1 weeks (2).

Perhaps the most useful frame of reference for risk stratification is the rate of extreme prematurity. Nulliparous women with twins delivered before 32 weeks 10.2% of the time and before 28 weeks 3.3% of the time. Corresponding rates were 11.8% and 4.0% for parous women with a prior preterm birth and 6.3% and 1.8% for parous women without a prior preterm birth (Table 3). Using the outcomes of this research, women undergoing IVF may be better counseled regarding their risk of a preterm twin delivery. This may ultimately lead to the integration of risk stratification based on a previous pregnancy to counsel women on the number of embryos to transfer at the time of IVF.

There are two strengths of this study related to the analysis of the SART CORS dataset. First, this study represents the use of IVF data to answer an obstetrical question. Second, precise gestational age is challenging to determine outside an IVF context. The SART database contains precise information about the date of conception and delivery. This analysis may encourage other investigators to use IVF data to study other obstetrical scenarios where precise pregnancy dating is important for the analysis.

Second, although not the main aim of this study, we did find vanishing gestations to be associated with higher rates of preterm delivery and low birth weight in the remaining twin pregnancies. In nulliparous women, the rate of preterm delivery of twins was 67.0% when 3 or 4 fetal heartbeats were initially seen compared with 60.6% when only two heartbeats were seen on initial ultrasound (Supplemental Table 1). These data correspond to increased preterm birth rates and low birth weight seen in singleton pregnancies after double embryo transfer or a vanishing cotwin (10, 11).

In conclusion, parous women carrying twins without a history of preterm delivery had lower rates of preterm delivery and low birth weight than did either nulliparous women or parous women with a history of prior preterm birth. Whereas the differences in rates are statistically significant, the magnitude of the differences is small compared with the differences in rates of preterm delivery and low birth weight between singleton and twin pregnancies in general.

## Figures and Tables

**Supplementary Figure:**