# Supplementary material for: Effects of parity on preterm delivery in twin gestations conceived with in vitro fertilization
Source: F S Rep. 2023 Jan 21;4(1):49–54. doi: 10.1016/j.xfre.2023.01.005 (PMC10028473; doi:10.1016/j.xfre.2023.01.005)
Supplement: Supplementary Table [file mmc1.docx]

**Supplemental Material**

**Supplemental Table 1: Evaluation of confounders to include in logistic regression analysis**

| subgroup analysis of nulliparous patients (n = 14,470) | | | | |
| --- | --- | --- | --- | --- |
|  | low birth weight < 2500g | p value | delivery prior to 37 weeks | p value |
| Age < 35 years, n = 7,353 | 56.8% | 0.99 | 61.8% | 0.006 |
| Age $\geq$35 years, n = 7,117 | 56.8% |  | 59.6% |  |
| Age continuous^*†^ | OR 1.0013 | 0.69 | OR 0.9938 | 0.06 |
|  |  |  |  |  |
| BMI < 25, n = 6,519 | 57.3% | 0.17 | 59.4% | < 0.01 |
| BMI $\geq$25, n = 5,378 | 56.1% |  | 63.5% |  |
| BMI continuous | OR 0.9982 | 0.58 | OR 1.0199 | < 0.01 |
|  |  |  |  |  |
| Female/female, n = 3,443^*^ | 62.3% | ref | 61.2% | ref |
| Female/male, n =7,285^*^ | 56.7% | <0.001 | 60.2% | 0.32 |
| Male/male, n = 3,742^*^ | 51.9% | <0.001 | 61.3% | 0.89 |
|  |  |  |  |  |
| Prior SAB, n = 9,777 | 56.8% | 0.96 | 60.9% | 0.39 |
| No prior SAB, n = 4,693 | 56.7% |  | 60.2% |  |
|  |  |  |  |  |
| Autologous oocytes, n = 11,733^*†^ | 56.1% | 0.002 | 59.9% | <0.001 |
| No autologous oocytes, n = 2,737 | 59.5% |  | 64.6% |  |
|  |  |  |  |  |
| Fresh embryo, n = 7,941 ^*†^ | 58.8% | <0.001 | 59.1% | <0.001 |
| No fresh embryo, n = 6,529 | 54.3% |  | 62.6% |  |
|  |  |  |  |  |
| Number of fetal heartbeats |  |  |  |  |
| 2, n = 14,164 ^*†^ | 56.6% | 0.003 | 60.6% | 0.02 |
| 3 or 4, n = 306 | 65.0% |  | 67.0% |  |
|  |  |  |  |  |
| PGT all or some embryos, n = 1,496 ^*†^ | 53.7% | 0.013 | 58.7% | 0.09 |
| No PGT, n = 12,974 | 57.1% |  | 60.9% |  |
|  |  |  |  |  |
| White, n = 6,580 ^*†^ | 54.8% | <0.001 | 61.6% | 0.04 |
| Other, n = 7,890 | 58.4% |  | 59.9% |  |
| Asian, n = 1,153^*†^ | 62.7% | <0.001 | 57.5% | 0.02 |
| Other, n = 13,317 | 56.3% |  | 61.0% |  |
| Hispanic, n = 686 | 58.6% | 0.32 | 61.5% | 0.66 |
| Other, n = 13,784 | 56.7% |  | 60.7% |  |
|  |  |  |  |  |
| Male factor, n = 4,796 ^*^ | 55.9% | 0.16 | 60.6% | 0.87 |
| No male factor, n = 9,674 | 57.2% |  | 60.8% |  |
| Diminished ovarian reserve, n = 3,913^*^ | 58.4% | 0.02 | 61.5% | 0.24 |
| No diminished ovarian reserve, n = 10,557 | 56.2% |  | 60.4% |  |
| Tubal factor, n = 2,111^*^ | 58.8% | 0.04 | 60.8% | 0.90 |
| No tubal factor, n = 12,359 | 56.4% |  | 60.7% |  |
| Unexplained infertility, n = 1,998^*†^ | 55.4% | 0.16 | 57.6% | 0.002 |
| No unexplained infertility, n = 12,472 | 57.0% |  | 61.2% |  |

Data are reported as percentages. P values are calculated by Chi-Square test for categorical data and univariate logistic regression for continuous variables. Unadjusted p values are reported. * indicates a predictor included in the final model for low average birth weight. ^†^ indicates a predictor included in the final model for preterm delivery. There were no changes between the preliminary and final models for preterm delivery or low average birth weight.

**Supplemental Table 2: Analysis of obstetrical outcomes based on parity number**

|  | para 1 | | | para 2 | | | para 3 or greater | | |
| --- | --- | --- | --- | --- | --- | --- | --- | --- | --- |
| total n = 16,266 | all  N = 12,214 | prior preterm birth  n = 1,338 | no preterm birth  n = 10,876 | all  n = 2,680 | prior preterm birth  n = 488 | no preterm birth  n = 2,192 | all  N = 1,372 | prior preterm birth  n = 253 | no preterm birth  n = 1,119 |
| Birth weight in grams (mean) | 2503 (532) | 2336 (591) | 2523 (520) | 2507 (525) | 2405 (520) | 2529 (523) | 2429 (564) | 2286 (610) | 2461 (548) |
| Birth weight in grams (median) | 2566 [2240, 2863] | 2410 [2013, 2736] | 2589 [2258, 2864] | 2566 [2254, 2849] | 2452 [2126, 2750] | 2594 [2268, 2863] | 2495 [2155, 2796] | 2353 [2000, 2693] | 2523 [2196, 2821] |
| Birth weight >4000g | 10 (0.1%) | 0 (0.0%) | 10 (0.1%) | 2 (0.1%) | 0 (0.0%) | 2 (0.1%) | 3 (0.2%) | 1 (0.4%) | 2 (0.2%) |
| Low birth weight, <2500g | 5,420 (44.4%) | 743 (55.5%) | 4,677 (43.0%) | 1,181 (44.1%) | 266 (54.5%) | 915 (41.7%) | 692 (50.4%) | 153 (60.5%) | 539 (48.2%) |
| Very low birth weight, <1500g | 609 (5.0%) | 122 (9.1%) | 487 (4.5%) | 118 (4.4%) | 24 (4.9%) | 94 (4.3%) | 99 (7.2%) | 29 (11.5%) | 70 (6.3%) |
| Gestational age in weeks (mean) | 36.0 (2.5) | 35.1 (3.0) | 36.1 (2.4) | 35.9 (2.5) | 35.4 (2.6) | 36.0 (2.5) | 35.5 (2.8) | 34.7 (3.2) | 35.7 (2.7) |
| Gestational age in weeks (median) | 36.7 [35.0, 37.7] | 35.9 [34.0, 37.1] | 36.7 [35.1, 37.7] | 36.6 [35.0, 37.6] | 36.0 [34.1, 37.3] | 36.6 [35.1, 37.7] | 36.3 [34.4, 37.3] | 35.6 [33.6, 36.9] | 36.4 [34.7, 37.4] |
| Delivery prior to 39 weeks | 11,989 (98.2%) | 1,323 (98.9%) | 10,666 (98.1%) | 2,613 (97.5%) | 481 (98.6%) | 2,132 (97.3%) | 1,336 (97.4%) | 247 (97.6%) | 1,089 (97.3%) |
| Delivery prior to 37 weeks | 6,809 (55.7%) | 931 (69.6%) | 5,878 (54.0%) | 1,543 (57.6%) | 323 (66.2%) | 1,220 (55.7%) | 871 (63.5%) | 194 (76.7%) | 677 (60.5%) |
| Delivery prior to 34 weeks | 1,828 (15.0%) | 328 (24.5%) | 1,500 (13.8%) | 400 (14.9%) | 109 (22.3%) | 291 (13.3%) | 276 (20.1%) | 72 (28.5%) | 204 (18.2%) |
| Delivery prior to 32 weeks | 822 (6.7%) | 160 (12.0%) | 662 (6.1%) | 178 (6.6%) | 48 (9.8%) | 130 (5.9%) | 138 (10.1%) | 38 (15.0%) | 100 (8.9%) |
| Delivery prior to 28 weeks | 248 (2.0%) | 57 (4.3%) | 191 (1.8%) | 51 (1.9%) | 12 (2.5%) | 39 (1.8%) | 41 (3.0%) | 14 (5.5%) | 27 (2.4%) |

Data are reported as mean (SD), median [IQR] or n (%).

**Supplemental Table 3: Analysis of obstetrical outcomes in pregnancies medically or spontaneously reduced to twins**

|  | nulliparous | parous | | |
| --- | --- | --- | --- | --- |
|  | n = 306 | all  n = 298 | prior preterm birth  n = 37 | no preterm birth  n = 261 |
| Birth weight in grams (mean) | 2204 (658) | 2366 (600) | 2180 (480) | 2401 (561) |
| Birth weight in grams (median) | 2324 [1786, 2665] | 2412 [1990, 2807] | 2197 [1885, 2509] | 2466 [2095, 2775] |
| Birth weight >4000g | 0 | 0 | 0 | 0 |
| Low birth weight, <2500g | 199 (65.0%) | 162 (54.4%) | 27 (73.0%) | 135 (51.7%) |
| Very low birth weight, <1500g | 46 (15.0%) | 24 (8.1%) | 5 (13.5%) | 19 (7.3%) |
| Gestational age in weeks (mean) | 35.0 (3.3) | 35.7 (2.7) | 35.2 (2.5) | 35.7 (2.7) |
| Gestational age in weeks (median) | 36.1 [33.6, 37.3] | 36.7 [34.7, 37.4] | 35.9 [33.3, 36.9] | 36.7 [34.9, 37.6] |
| Delivery prior to 39 weeks | 299 (97.7%) | 294 (98.7%) | 35 (94.6%) | 259 (99.2%) |
| Delivery prior to 37 weeks | 205 (67.0%) | 179 (60.1%) | 29 (78.4%) | 150 (57.5%) |
| Delivery prior to 34 weeks | 85 (27.8%) | 52 (17.4%) | 10 (27.0%) | 42 (16.1%) |
| Delivery prior to 32 weeks | 56 (18.3%) | 25 (8.4%) | 4 (10.8%) | 21 (8.0%) |
| Delivery prior to 28 weeks | 18 (5.9%) | 8 (2.7%) | 0 | 8 (3.1%) |

Data are reported as mean (SD), median [IQR] or n (%).
